# Comparison of anti-CD3 and anti-CD28-coated beads with soluble anti-CD3 for expanding human T cells: Differing impact on CD8 T cell phenotype and responsiveness to restimulation

**DOI:** 10.1186/1479-5876-8-104

**Published:** 2010-10-26

**Authors:** Yixin Li, Roger J Kurlander

**Affiliations:** 1 Department of Laboratory Medicine, NIH Clinical Center, National Institutes of Health, Bethesda, Maryland, USA

## Abstract

**Background:**

The ability to expand virus- or tumor-specific T cells without damaging their functional capabilities is critical for success adoptive transfer immunotherapy of patients with opportunistic infection or tumor. Careful comparisons can help identify expansion methods better suited for particular clinical settings and identify recurrent deficiencies requiring new innovation.

**Methods:**

We compared the efficacy of magnetic beads coated with anti-CD3 and anti-CD28 (anti-CD3/CD28 beads), and soluble anti-CD3 plus mixed mononuclear cells (designated a rapid expansion protocol or REP) in expanding normal human T cells.

**Results:**

Both anti-CD3/CD28 beads and soluble anti-CD3 promoted extensive expansion. Beads stimulated greater CD4 cell growth (geometric mean of 56- versus 27-fold (p < 0.01) at day 21) but both stimulated similar CD8 expansion (189- versus 186-fold). Phenotypically, bead-treated CD4 and CD8 T cells and anti-CD3-treated CD4 cells typically assumed an effector/effector memory phenotype by day 14. By comparison, a subset of anti-CD3-treated CD8 cells, derived from naïve cells, retained much greater expression of CD45RA, CD27 and CCR7, than matched bead-treated cells despite comparable expansion. These cells were clearly distinguishable from CD45RA+ terminally differentiated effector cells by the presence of CD27, the absence of CD57 and their inability to produce cytokines after stimulation. When used to expand previously stimulated cells, anti-CD3 plus autologous MNCs produced much less antigen-induced cell death of CD8 cells and significantly more CD8 expansion than beads.

**Conclusions:**

Anti-CD3/CD28 beads are highly effective for expanding CD4 cells, but soluble anti-CD3 has significant potential advantages for expanding CD8 T cells, particularly where preservation of phenotypically "young" CD8 cells would be desirable, or where the T cells of interest have been antigen-stimulated in vitro or in vivo in the recent past.

## Background

With advances in the methods for selecting and manipulating T cells there is increasing interest in the adoptive transfer of bioactive T cells as a treatment for infections and cancer. This approach has been used successfully to transfer antiviral immunity after stem cell transplantation [1], and is under active investigation in treating malignancy [2]. Antigen-specific T cells suitable for transfer can only be retrieved from blood or tissue sites in relatively small numbers, consequently they usually are expanded specifically or nonspecifically prior to transfer. Such ex vivo manipulations, however, potentially can damage T cell homing, proliferation, and survival after infusion [3,4]. Given this risk, the choice of methods may have important implications for clinical efficacy.

Antibodies against CD3 are a central element in many T cell proliferation protocols. Immobilized on a surface, anti-CD3 delivers a strong proliferative signal through the T cell receptor complex (signal 1) but in the absence of additional costimulatory signals (signal 2), the resulting proliferation is often followed by premature T cell apoptosis or anergy [5]. By immobilizing anti-CD3 and anti-CD28 to simultaneously deliver signal 1 and a costimulatory signal 2, proliferation can be increased without provoking early cell death [6]. The expanding cells also demonstrate enhanced ability to release cytokines and lyse targets cells in an MHC unrestricted manner [7]. Consequently, magnetic beads coated with anti-CD3 and anti-CD28 (anti-CD3/CD28 beads) have proved a convenient reagent for expansion which has been used experimentally to boost T cell immunity in immunosuppressed cancer patients [8-10] and enhance the anti-tumor effect of donor lymphocyte infusions after allotransplantation [11]. These studies have established that beads can be used to expand functional T cells, and that some of these cells can persist in vivo postinfusion.

While these results are encouraging, the bead expansion technique has limitations. Ex vivo expansion stimulates the generation of effector T cells with increased lytic and cytokine producing capability [7], but the capacity of these cells for additional homing and proliferation after infusion is uncertain [3]. While CD4 cells respond very well to anti-CD3/CD28 stimulation, CD8 cells proliferate less extensively with an increased rate of apoptosis [12]. Given the importance of CD8 T cells in the anti-tumor response, this is a significant concern.

One commonly used alternative approach for stimulating proliferation is the incubation of T cells with soluble anti-CD3 antibody in the presence of Fc receptor bearing accessory cells [13-15], an approach designated the "Rapid Expansion Protocol" (REP). Antibody "presented" to T cells in this manner clearly generates a more effective proliferative signal than soluble anti-CD3 alone or anti-CD3 immobilized on a plastic surface [16]. This presumably reflects the dual benefit of more extensive anti-CD3-T cell receptor crosslinking on a surface, and the costimulation provided by cell-cell interaction between T cells and Fc receptor positive accessory cells such as monocytes which constitutively express CD80 [17], CD86 [17], and CD137 [18]. These complex interactions in some respects mimic events during physiologic antigen presentation. Given its efficacy, this approach has been used extensively for expansion of T cell clones and lines for in vitro and clinical adoptive transfer studies [13-15,19].

To gain further insight into the similarities and differences between the T cell responses produced by beads and REP, the current studies critically compare their impact on T cell survival, proliferation, and phenotype. While both beads and anti-CD3 are effective in expanding T cells, our studies demonstrate substantial differences in their impact on CD8 cells that merit consideration in situations where preservation of the CD8 T cell response in important.

## Methods

### Antibodies, beads, and chemicals

CD45RA/FITC, CD45RA/PE, CD57/FITC, CD28/PE, CD4/PerCP, CD8/PerCP, CD27/APC, brefeldin A, anti-IFNγ/FITC, anti-TNFα/PE, 7-Amino-Actinomycin D (7-AAD), and appropriate isotype controls were purchased from BD Biosciences. Anti-human CCR7-phycoerythrin was obtained from R&D Systems. Biotinylated anti-CD3 and anti-CD28 antibodies were purchased from eBioscience. Anti-CD3 (Orthoclone OKT3) was provided by Stephen Migueles (NIAID, Bethesda, MD). Flow-Check Fluorospheres were purchased from Beckman Coulter. Streptavidin-labeled Dynabeads (M280) and CD3/CD28 T cell expander beads were obtained from Invitrogen. Carboxyfluorescein succinimidyl ester (CFSE) was purchased from Molecular Probes (Eugene, OR) and recombinant human IL-2 was purchased from PeproTech (Rocky Hill NJ).

### Preparation of anti-CD3/CD28 beads

To prepare antibody-coated beads of varying composition, streptavidin-labeled beads were coated with varying mixtures of biotinylated anti-CD3 and anti-CD28 antibodies. To this end, streptavidin-M280 beads were washed once with sterile PBS/BSA and resuspended at 10-50 millions beads/ml. Preliminary dose response studies, using FITC-labeled anti-mouse IgG and flow cytometry to monitor biotinylated antibody binding to beads, established that beads were saturated by 100 ng of biotinylated antibody/million beads. Consequently this total immunoglobulin/bead ratio was routinely used for bead coating. To vary the ratio of antibody coating on beads equimolar solutions of anti-CD3 and anti-CD28 were mixed at 1:0, 1:5, 1:10, 1:20, 1:40, 1:80, 2 1:160, and 0:1 ratios. Control beads were coated with biotinylated IgG1 isotype. Coating was performed on a rotator stand at room temperature for 2-3 hours. Beads were then washed two times with filtered PBS/BSA, once with complete medium, and then resuspended in RPMI 1640 complete medium. Antibody coating was performed as needed, but preliminary studies established that beads could be stored 4°C for at least one week without any change in potency. In selected studies, T cells were also stimulated using commercially prepared anti-CD3 and anti-CD28 coated beads (CD3/CD28 T cell Expander, Invitrogen).

### Flow cytometry

Flow cytometry was performed using a 4-color Facscalibur (BD biosciences). The standard phenotypic analysis was performed at different time point using antibody panels as described in the results. The flow data was analyzed using Flow-Jo software.

### Human leukocyte acquisition and purification

Normal healthy donors gave informed consent to blood donation or leukapheresis procedures performed as specified in clinical protocols approved by the Institutional Review Board of the Clinical Center of the National Institutes of Health. Mixed mononuclear cells (MNCs) obtained by leukepheresis or prepared from buffy coats using Ficoll-Paque density gradient centrifugation, were cryopreserved, and thawed as previously described [20].

To prepare T cell subsets for selected experiments, MNCs from freshly collected buffy coat cells were purified by negative selection using CD8^+ ^Memory T Cell Isolation and CD8^+ ^Naive T Cell Isolation Kits purchased from Miltenyi Biotech.

### Monitoring T cell division and early expansion using CFSE labeled cells

To monitor cells division and expansion during the early days after stimulation, cells were CFSE-labeled and monitored using methods described by Hawkins, et al. [21]. In brief, to label cells, 2-5 × 10^7 ^mixed mononuclear cells or cultured T cells maintained in RPMI 1640 containing 10% fetal calf serum plus 100 unit/ml penicillin, 100 ug/ml streptomycin, and 2 mM glutamine (RPMI/FCS) were incubated with 2 μM CFSE at 37°C for 10 min. Cells were then washed three times to remove unbound CFSE, resuspended in fresh RPMI/FCS, and incubated overnight. Labeled cells were then distributed (50,000/well) in a 96 well round bottom plate in wells also containing anti-CD3/CD28 coated beads (three beads/cell), anti-CD3 (30 ng/ml), or no additional stimulator. When using anti-CD3 to re-treat previously stimulated cells, 100,000 irradiated MNCs (accessory cell:responder ratio of 2:1) were also added as a source of Fc receptor positive accessory cells suitable for "presentation" of anti-CD3 to T cells. Fresh cells received IL2 (50 U/ml) on day 2. Restimulated cells were maintained with 50 U/ml of IL2 from day 1. Wells were fed with additional medium containing IL2 at day 4 or 5 and every 2-3 days thereafter. With continued growth, the contents of wells were diluted 4 fold into new wells with fresh medium and IL2 as needed to prevent overcrowding.

To monitor cell growth, at selected time points after stimulation, 10,000 calibrator beads/well (Flow-Check Fluorospheres, Beckman Coulter, CA) were added to wells along with PE labeled anti-CD4 or anti-CD8 antibodies. The contents of the well were mixed, incubated for one half hour, and then washed twice with PBS/BSA (1%) before addition of 7-AAD to exclude dead cells in flow cytometry analysis. All measurements of cell composition and number were performed in quadruplicate.

The absolute number of cells per well at each time point was calculated based on the number of calibrator beads and the number of viable cells detected per well by flow cytometry using the formula:

The proportion of cells undergoing 0-6 divisions could then be quantitated based on the pattern of CFSE fluorescence using Flo-Jo software, and the total number of viable cells per well.

### Bulk stimulation of T cells in vitro

To monitor cell phenotype and cell expansion over a 3 week period, fresh MNC or cells expanded previously using anti-CD3/CD28 beads or anti-CD3 were cultured in 12 well plates (5 × 10^6 ^cells in 2 ml/well) with anti-CD3/CD28 beads (three beads/cell), anti-CD3 (30 ng/ml), or medium alone. Previously expanded cells restimulated with anti-CD3 routinely also received irradiated autologous MNC (2 cells/responder) as a source of Fc receptor positive accessory cells. Medium containing recombinant human IL2 (50 units/ml) was added to freshly cultured cells at day 2 and to restimulated cells throughout the process. Beads were removed using a magnet on day 7 post stimulation. Cell counts of freshly stimulated cells were monitored at least twice weekly and cultures were fed every other day with fresh RPMI/FCS and IL2, and transferred to flasks or frozen as needed to maintain cell numbers between 0.75 and 2 × 10^6^/ml. Because of the presence of irradiated autologous feeder cells in REP treated cells, viable cell counts were not used to monitor cell growth in restimulated cultures until after day 7 by which time no more viable irradiated cells were present.

### Measurement of Intracytoplasmic cytokine Production

T cells harvested 14 days after stimulation with anti-CD3/CD28 beads or soluble anti-CD3 were treated for 4 hours with phorbol myristate acetate (PMA, 35 nM) and the calcium ionophore A23187 (0.5 μM) or with medium alone in the presence of brefeldin (Golgiplug, BD Bioscience). Cells were then incubated with anti-CD8 PerCP and CD27 APC for 30 minutes, fixed and permeabilized using Cytofix/cytoperm solution (BD Bioscience) as recommended by the manufacturer, and stained intracellularly using anti-IFNγ FITC and antiTNFα PE. Duplicate samples were stained with an appropriate isotype control. Cytokine expression in treated and control cells was then assessed using flow cytometry.

### Statistics

Paired t-tests and nonparametric 2-tail Wilcoxon matched pairs tests were performed using Graphpad Prism Software.

## Results

### Time course for T cell response to stimulation

**Figure 1:**
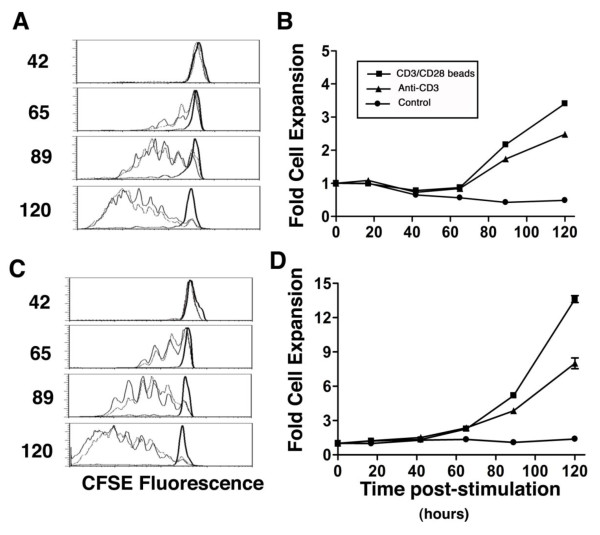
**Early time course of T cell division and expansion in response to anti-CD3/CD28 beads and soluble anti-CD3**. CFSE-labeled CD4 (A) and CD8 (C) cells began dividing 40-60 hours after exposure to beads (solid lines) or anti-CD3 (dashed lines), but divided minimally in the absence of stimulation (2× solid lines). The number of stimulated and control CD4 (B) and CD8 (D) T cells remained comparable until about 60 hours after stimulation when measureable cell expansion began.

The CFSE-labeled CD4 and CD8 T cells in MNCs began dividing 40-50 hours after stimulation with anti-CD3/CD28 beads or soluble anti-CD3. CD8 cells divided slightly more rapidly than CD4 cells, and there were no consistent differences in early response to the two stimuli (Figure 1A and 1C). The number of viable cells at hour 40 (just before proliferation began) was similar in unstimulated, bead-stimulated, and anti-CD3-stimulated wells indicating neither stimulus caused extensive early activation-induced cell death (AICD) (Figure 1B and 1D). Consistent with the timing of cell division, expansion in cell number in response to either stimulus was delayed until 50-60 hours after stimulation.

**Figure 2:**
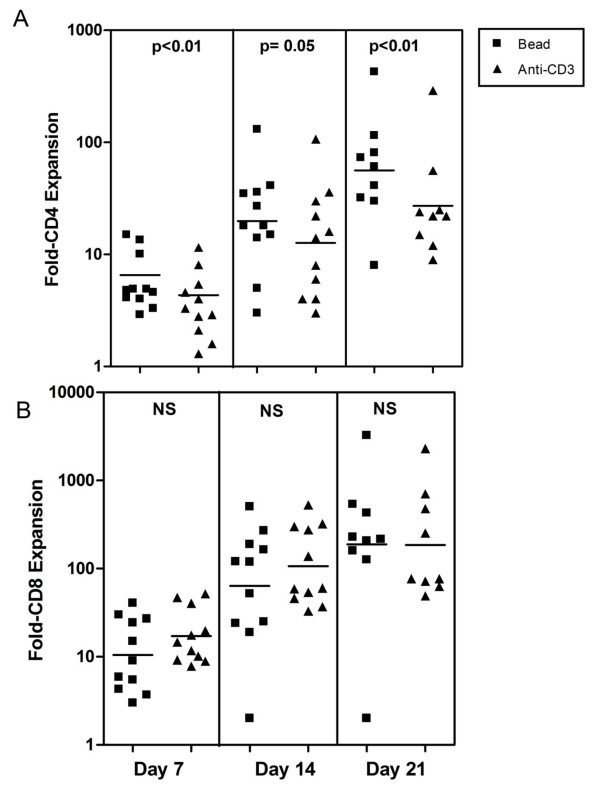
**Comparison of CD4 (A) and CD8 (B) expansion after stimulation with anti-CD3/CD28 beads (squares) or anti-CD3 (triangles)**. Beads stimulated significantly greater CD4 expansion than anti-CD3 (p values for statistical significance at each time point is indicated at the top of each box). CD8 expansion was slightly greater in response to anti-CD3 at 7 and 14 days, but this trend was not statistically significant. The p values were calculated using the Wilcoxon matched pairs test.

To compare the expansion produced by anti-CD3/CD28 beads and anti-CD3, we monitored changes in cell number in bulk cultures over a 21-day period in 11 separate studies (Figure 2) and noted several persistent trends. Consistent with the more rapid rate of early cell division noted in Figures 1A and 1C, CD8 cells expanded more rapidly than CD4 cells in response to either stimulus. Focusing on CD4 cells, this subset expanded more rapidly in response to beads than anti-CD3 (Figure 2A) and this difference was statistically significant at days 7, 14, and 21. There was a trend to more rapid expansion of CD8 cells in response to anti-CD3, particularly at days 7 and 14, but these differences did not achieve statistical significance (Figure 2B). Consistent with these reciprocal trends in CD4 and CD8 expansion, cultures stimulated with anti-CD3 accumulated a significantly higher proportion of CD8 cells at all three time points than matched bead-treated cultures (Table 1).

**Table 1:** % CD8 T cells in primary cultures at 7-21 days after stimulation with anti-CD3/CD28 beads and OKT3

	**Day after Stimulation**		
	
**Cells stimulated with:**	7	14	21
	
Anti-CD3/CD28 beads	34.0 ± 6.8 (11)*	47.6 ± 6.3 (11)	48.9 ± 5.4 (9)
OKT3-treated	53.7 ± 7.0 (11)	68.0 ± 5.4 (11)	63.3 ± 4.4 (9)
	P < 0.005	P < 0.005	P < 0.05

*The results represent the mean ± SE for matched pairs of cultures with the number of experiments marked in parentheses. Paired t-test was used to assess significance.

These studies were performed with beads coated with anti-CD3 and anti-CD28 at a ratio of 1:20 but similar results were obtained using beads coated at ratios of 1:5, and 1:80 and with commercially available T cell expander beads (data not shown). Comparisons of expansion produced by anti-CD3 at 30 and 300 ng/ml also yielded essentially identical results (data not shown).

### Phenotypic changes in T cells during in vitro expansion

**Figure 3:**
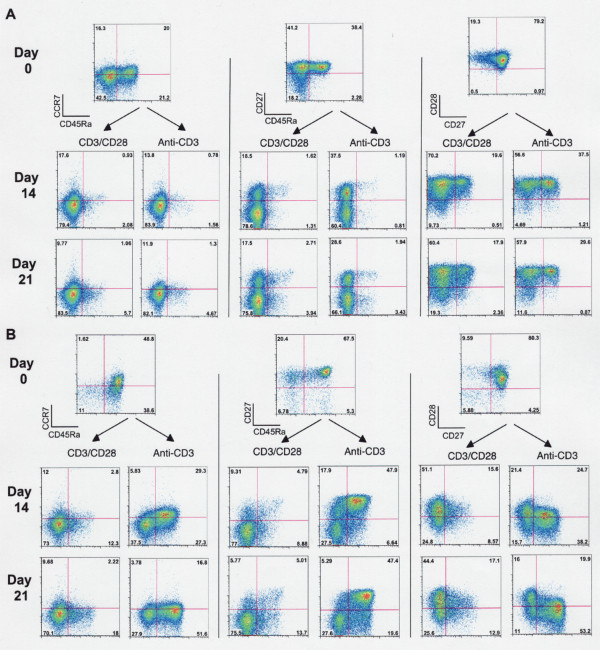
**Comparison of T cell phenotype 14 and 21 days after primary stimulation of MNCs with CD3/CD28 beads and soluble anti-CD3**. CD4 T cells (A) showed a similar pattern of changes in response to either stimulus, but CD8 cells (B) demonstrated significant differences in phenotype. At comparable levels of expansion, anti-CD3 treated CD8 cells retained higher levels of CD45RA, CD27, and CCR7 expression than anti-CD3/CD28 bead treated cells and bead-treated CD8 cells expressed higher levels of CD28.

Peripheral blood T cells are usually subclassified as naïve (CD45RA+, CCR7+), central memory (CD45RA-, CCR7+), effector memory (CD45RA-, CCR7-), or effector cells (CD45RA+, CCR7-)[22]. Naïve cells also express CD27 and CD28, which are both progressively lost with post-thymic proliferation and "differentiation" towards an effector phenotype [23-26]. To compare the impact of beads and anti-CD3, the expression of these markers on CD4 and CD8 cells was assessed before and after 2-3 weeks of in vitro stimulation. The results from one representative experiment are illustrated in Figure 3.

The phenotype of CD4 T cells was similarly affected by exposure to beads or anti-CD3 (Figure 3A) with substantial reductions in the expression of CD45RA, CCR7, and CD27. CD8 T cells, on the other hand, were affected differently by beads and anti-CD3. By day 14, CD45RA expression on bead-treated cells was markedly reduced, but a substantial fraction of anti-CD3-treated cells retained CD45RA expression (Figure 3B). This subpopulation was strongly CD27 positive, and (to a lesser extent) CCR7 positive. The same pattern of CD45RA and CD27 expression was seen at day 21, but CCR7 expression often diminished substantially by this time point. On the other hand, bead-treated cells usually retained a higher proportion of CD28 positive cells both at day 14 and 21. The size of the CD45RA+, CD27+ subset of CD8 cells in anti-CD3-treated cultures varied somewhat from donor to donor, but the underlying pattern was qualitatively consistent.

**Figure 4:**
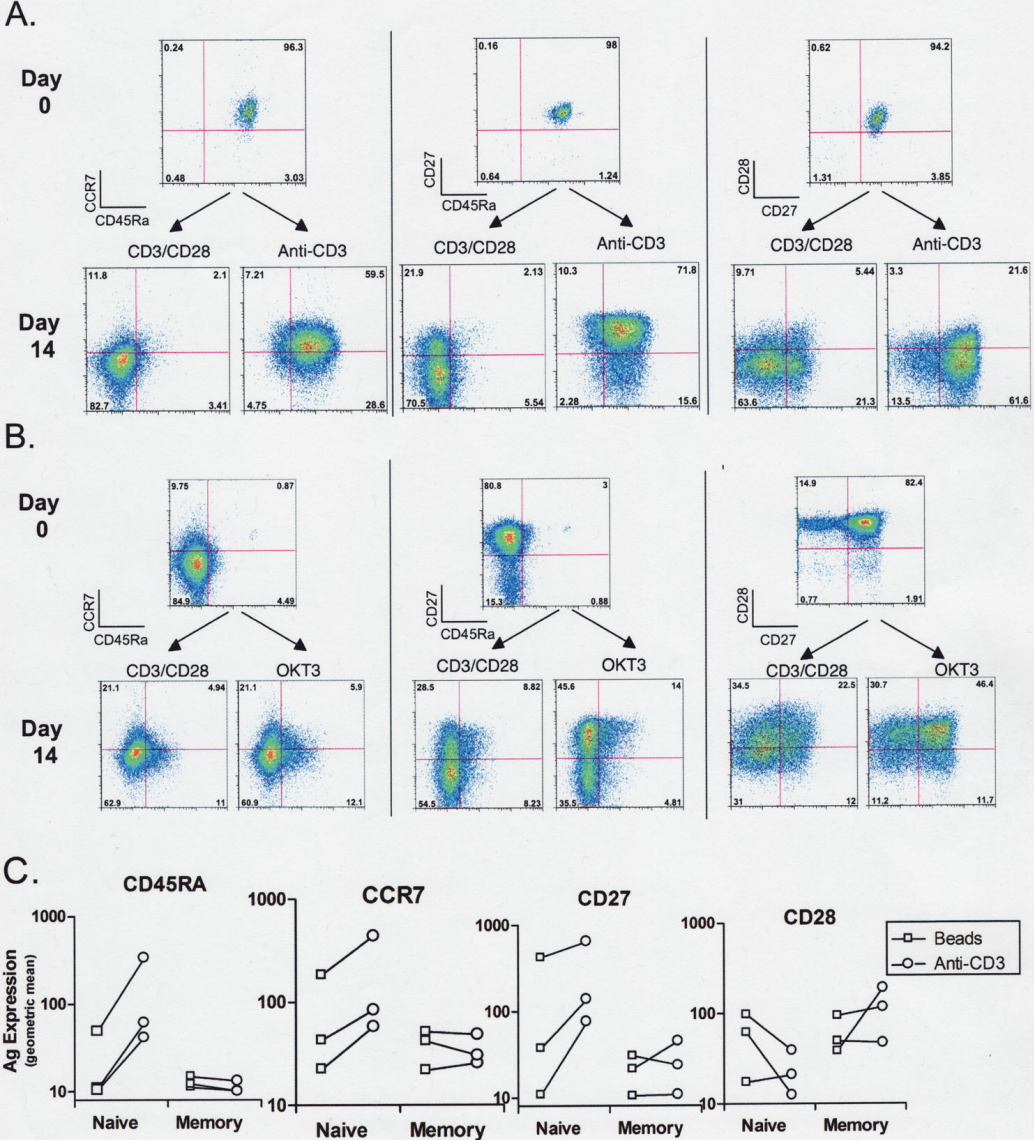
**Comparison of the impact of anti-CD3/CD28 beads and soluble anti-CD3 plus irradiated MNCs on expansion by purified naive (A) and memory (B) CD8 cells**. Significant differences in the phenotype of expanded naïve T cells, but not in memory cells, were noted. Panel C collates the results of 3 experiments quantitating changes in CD45RA, CCR7, CD27, and CD28 surface antigen (expressed as geometric mean channel fluorescence) when naïve and memoryCD8 cells were expanded using beads (squares) or anti-CD3/MNCs(circles) for 14 days.

To clarify the origin of the persistent CD45RA+/CD27+ subset, we stimulated purified naïve and memory CD8 populations with anti-CD3/CD28 beads or anti-CD3 plus irradiated MNCs as a source of FcR+ accessory cells. A substantial proportion of anti-CD3-treated naïve CD8 cells maintained the CD45RA+, CCR7+, CD27+ phenotype noted above, but much fewer bead-treated cells demonstrated this phenotype (Figure 4A and 4C). Memory cells did not consistently differ in their pattern of CD45RA and CD27 retention in response to either stimulus (Figure 4B and 4C). Thus the phenotypic differences noted in mixed populations can be largely attributed to variations in the response of naïve CD8 T cells to soluble anti-CD3 plus accessory cells versus beads.

**Figure 5:**
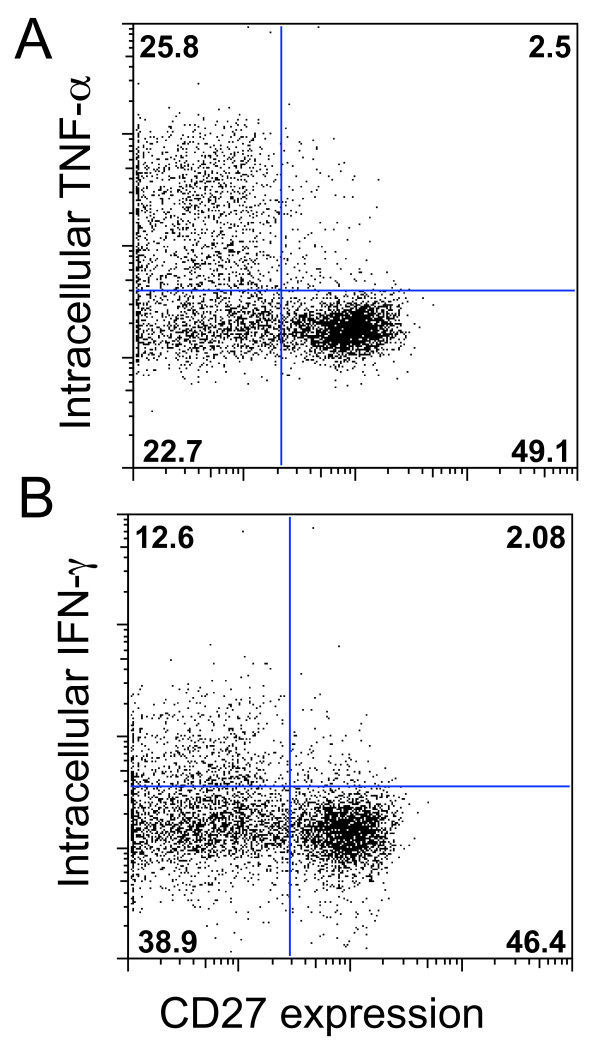
**Intracytoplasmic cytokine production by day 14 anti-CD3 stimulated CD8 T cells stimulated for 4 hours with PMA/A23187**. By comparison to CD27- cells, the CD27+ subset produced little intracytoplasmic (A) tumor necrosis factor (TNF-α) or (B) interferon- γ (IFN-γ). Similar results were obtained in each of 4 studies.

CD45RA expression on cultured T cells is a typical characteristic of terminally differentiated effector T cells, but unlike conventional effectors [22], the CD45RA+ CD8 T cells noted after anti-CD3 treatment were consistently CD27+ (Figure 3) and CD57- (data not shown). Effector T cells typically produce intracellular cytokines within 4 hours after stimulation in vitro [22], but the CD27+ anti-CD3-expanded CD8 cells (in contrast with the CD27- cells in the same preparation) produced little intracellular IFNγ or TNFα in response to PMA/A23187 stimulation (Figure 5).

### T cell response to restimulation

**Figure 6:**
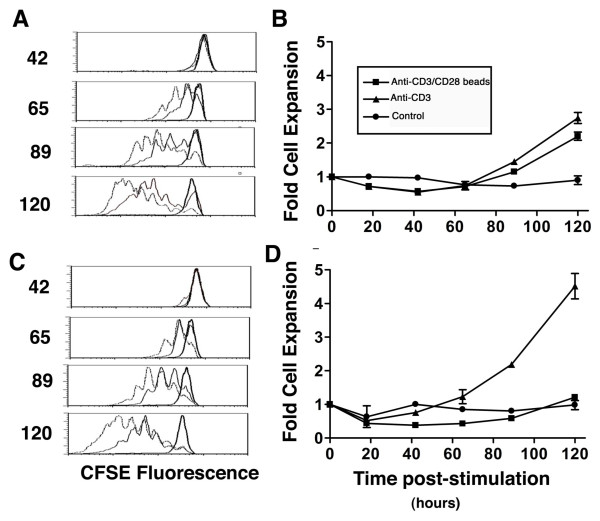
**Early time course for expansion of bead-expanded T cells after restimulation 14 days later with anti-CD3/CD28 beads or anti-CD3 plus irradiated MMCs**. Restimulated CD4 (A) and CD8 (C) both divided more extensively in response to anti-CD3 (dashed lines) than anti-CD3/CD 28 (solid lines), but this difference was more pronounced for CD8 cells. CD4 T cell expansion (C) was similar in response to either stimulus, but CD8 T cells (D) expanded substantially more rapidly than anti-CD3/CD28 bead-treated cells.

**Figure 7:**
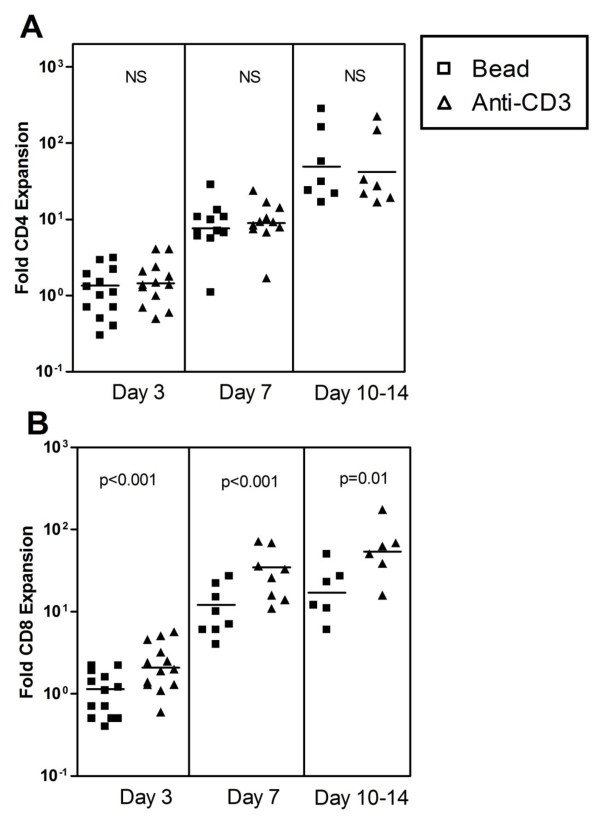
**Comparison of CD4 (A) and CD8 (B) growth when bead-treated T cells were restimulation with anti-CD3/CD28 beads (squares) or anti-CD3/irradiated MMCs (triangles)**. T cell expansion was monitored using CFSE labeled cells and flow cytometry as described in the methods. CD4 expansion was similar in response to either stimulus. By contrast, bead-treated CD8 cells expanded significantly less well than anti-CD3-treated cells at all time points. The p values with obtain using the Wilcoxon matched pairs test are indicated at the top of each box.

On occasion, the number of cells generated by one cycle of T cell expansion may be insufficient for the desired purpose, and further expansion would be desirable. To compare impact of restimulation, cells previously expanded using anti-CD3/CD28 beads were CFSE-labeled 6 to 63 days later, and restimulated with fresh anti-CD3/CD28 beads, anti-CD3 plus irradiated autologous MNCs, or, as a control, maintained in medium plus IL2 alone. Like fresh cells, restimulated cells showed an increased rate of division 40-50 hours post stimulation (Figure 6A and 6C). Unlike fresh cells (Figure 1B and 1D), however, restimulated cells (particularly those retreated with beads) often decreased in number relative to control cells over the first 40 hours of culture reflecting early AICD (Figure 7B and 7D).

The findings from 11 experiments comparing T cell expansion 3-14 days after restimulation are summarized in Figure 7. Despite the initial AICD in many cases, CD4 cells incubated with either stimulus expanded 10-100 fold by day 7 (figure 7A). CD8 T cells incubated with soluble anti-CD3 plus irradiated MNCs demonstrated a similar pattern, but bead-treated CD8 cells showed significantly less expansion at all 3 time points (Figure 7B).

**Figure 8:**
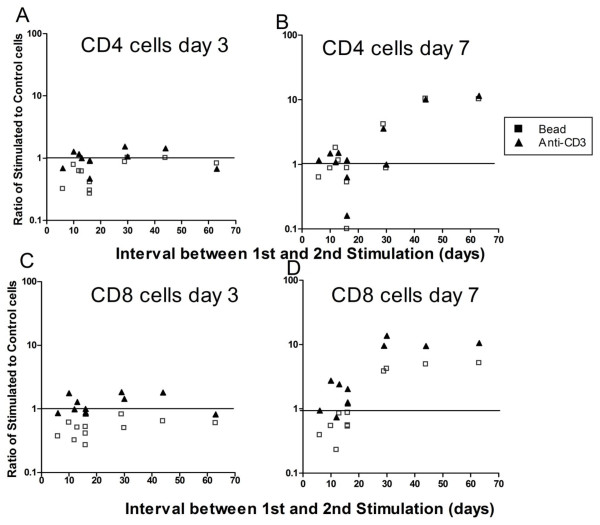
**Evaluation of the interrelationship between the type and timing of the second stimulus and expansion of restimulated CD4 (A and B) and CD8 (C and D) T cells**. To normalize for the impact of persistent growth attributable to primary stimulation, the ratio of growth after restimulation to growth by matched control cells in the absence of restimulation was calculated. Regardless of the interval between stimulations, by day 3, CD4 (A) and CD8 (B) cells re-incubated with anti-CD3/CD28 beads usually had a stimulation ratio of less than 1 i.e. cells had diminished in number compared to control untreated cells. Even at day 7 (B and D), the stimulation ratio seldom exceeded 1 for cells which had been rested in vitro between stimulations for less than 20 days. By comparison, the stimulation ratio for anti-CD3/MMC treated cells seldom dropped below 1 at day 3, and increased further with time, even when the rest interval between stimulations was less than 14 days.

Although the gross expansion of restimulated cells in some experiments (Figure 7) was comparable in magnitude to that observed after primary expansion (Figure 2), matched control cells also often expanded as well. To distinguish true restimulation-dependent growth from persistent expansion still attributable to primary stimulation, we calculated the ratio of expansion by stimulated cells/expansion by matched control cells and plotted this as a function of the time interval between first and second stimulation (Figure 8A-D). These plots make two important points. First, the expansion ratio for cells re-exposed to beads was almost always less than 1 at day 3 poststimulation (Figure 8A and 8C) reflecting AICD, and this effect was particularly severe for CD8 cells. By comparison soluble anti-CD3-treated cells seldom demonstrated this degree of early cell loss. Second, early restimulation (less than 20 days after primary stimulation) was associated with greater early cell loss at day 3, and poor expansion at day 7 (Figure 8B and 8D). This was most striking for bead-treated cells (particularly CD8 cells), but soluble anti-CD3 stimulated cells showed a similar, albeit less marked, trend. In these studies, only cells rested > 30 days between stimulations demonstrated substantial expansion relative to control cells. In sum, these studies emphasize that even when gross expansion is observed, restimulation (particularly with beads) may actually impede cell expansion.

While figures 5, 6, 7 focused on the responses of cells initially expanded using anti-CD3/CD28 beads, analogous studies performed using cells initially expanded using soluble anti-CD3 gave qualitatively identical results.

### Comparison of T cell size after stimulation with anti-CD3/CD28 beads or anti-CD3

**Figure 9:**
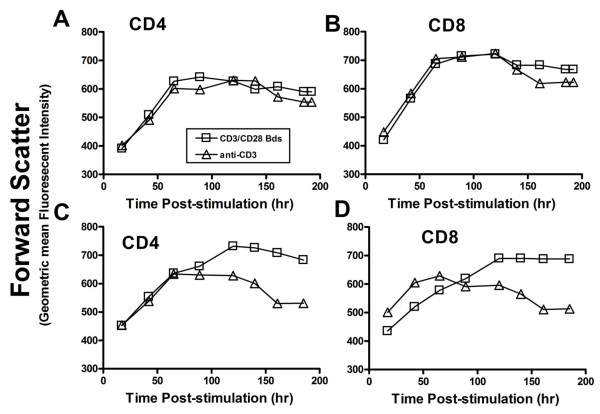
**Comparison of changes in forward scatter measured by flow cytometry (a measure of cell size) in response to anti-CD3/CD28 beads and anti-CD3**. Primary CD4 (A) and CD8 cells (B) showed similar changes in forward scatter after exposure to either stimulus, but CD4 (C) and CD8 (D) cells restimulated on day 30 increased in size more slowly and remained large for longer after restimulation with beads. This difference in size was noted even when the enlarged cells were expanding poorly.

To gain additional insight into the relative impact of beads and anti-CD3 on cells, we serially monitored forward scatter (a relative measure of cell size) in CD4 and CD8 cells after stimulation and restimulation. CD4 and CD8 T cell size increased in a similar manner after primary treatment with either stimulus (Figure 9A and 9B). Cells restimulated with anti-CD3/CD28 beads however increased in size more slowly and maintained a larger size for a longer period than matched anti-CD3 treated cells, even when they were expanding poorly. These differences in size could not be simply explained by differences in IL2 feeding schedule or cell concentration within flasks.

### Impact of variations in bead coating with anti-CD3 and CD28 on T cell responses to restimulation

**Figure 10:**
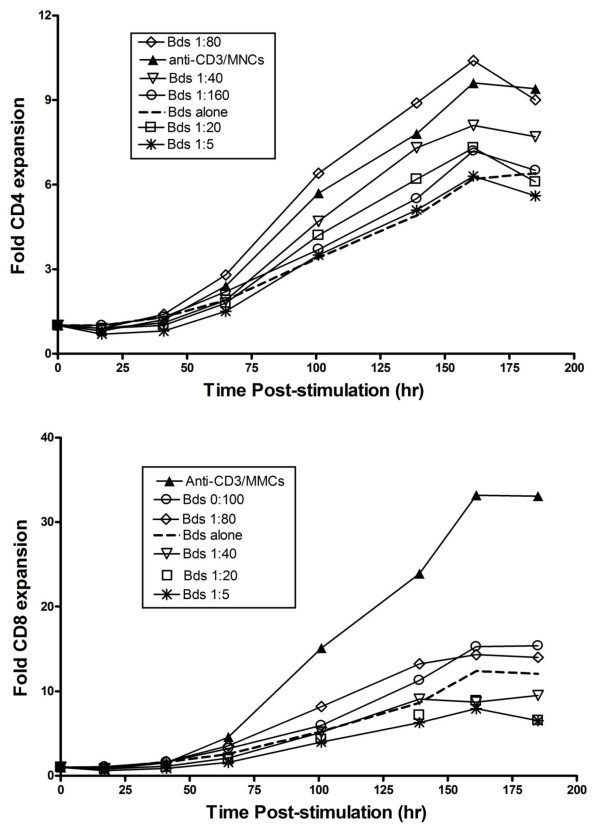
**Impact of variation in the anti-CD3:anti-CD28 coating ratio on the efficacy of anti-CD3/CD28 beads in restimulating previously expanded CD4 (upper panel) and CD8 (lower panel) T cells**. CD4 cells expanded most extensively in response to soluble anti-CD3 plus irradiated MMCs or beads coated at a very low anti-CD3:anti-CD28 ratio. Expansion was inhibited by higher levels of anti-CD3 coating. CD8 cells also responded better to beads coated at a low anti-CD3:anti-CD28 ratio, but even at the lowest ratio tested, beads were considerably less effective than anti-CD3/MMCs in promoting cell growth.

To assess whether bead-mediated expansion could be improved by modifying the ratio of anti-CD3 to anti-CD28 coating, we restimulated cells with beads coated using a variety of antibody ratios (Figure 10). Restimulated CD4 cells expanded better (overlapping in efficacy with anti-CD3 plus irradiated MNCs) in response to beads coated using lower anti-CD3: anti-CD28 ratios. CD8 cell expansion was also improved by reducing the anti-CD3:anti-CD28 ratio but even using the most lightly coated beads (or beads coated with anti-CD 28 alone), expansion remained substantially inferior to that produced using anti-CD3/MNCs. The poor response of CD8 cells to beads was not appreciably improved by adding irradiated MNCs and sufficient beads to maintain a 3:1 bead to total cell ratio (data not shown).

## Discussion

Anti-CD3/CD28 beads and soluble anti-CD3 both stimulate extensive polyclonal expansion of human peripheral blood T cells. Beads show a small but significant advantage in expanding CD4 cells and anti-CD3 demonstrates a trend towards more rapid early CD8 cell expansion. Proliferation of anti-CD3 treated cells slows after day 14, while bead-mediated proliferation typically continues for more than 21 days. Judged solely by their efficacy in promoting expansion, beads are more effective. Our studies, however, identify two qualitative differences that may merit consideration in tailoring an expansion method for any particular clinical situation.

First, beads and anti-CD3 have quite different effects in restimulating CD8 T cells. AICD, mediated at least in part through Fas/FasL interaction and activation of the proapoptotic molecule BIM, is a well-described complication of T cell stimulation [27,28]. Although CD28 costimulation enhances expression of the anti-apoptotic molecule BCL-X_L_, concurrent anti-CD3 and anti-CD28 signaling can promote CD8 apoptosis [12]. The current studies demonstrate that previously stimulated CD8 cells are particularly susceptible to AICD and growth retardation after bead exposure. This effect is not limited to cells previously exposed to beads. Cells initially stimulated using soluble anti-CD3 or PHA (data not shown) respond in the same manner. This sensitivity persists even in cells rested for more than 3 weeks between stimulations. This was not an isolated observation using one particular bead formulation. Similar results were observed using commercially available beads and "home-brew" beads anti-CD3 and anti-CD28 coated at a variety of ratios.

Soluble anti-CD3 used in conjunction with irradiated MNCs to restimulate cells produced significantly less AICD and more CD8 T cell growth. The difference was particularly striking when CD8 T cells were retreated before cells had "rested" sufficiently after primary stimulation. While anti-CD3 might fail to increase the growth rate of still expanding cells, it did not produce the striking AICD and extended growth retardation associated with anti-CD3/CD28 beads.

The mechanism underlying this difference was not addressed in these studies, but a variety of factors may contribute. Judging by the differences in time course for changes in cell size after restimulation (Figure 9), soluble anti-CD3 generates a less pronounced and prolonged T cell perturbation in restimulated cells than anti-CD3/CD28 beads. Equally important, Fc receptor bearing monocytes "presenting" anti-CD3 to T cells, express not only the CD28 ligands CD80 and CD86, but CD137L which can activate CD137 [18], another potent costimulatory molecule for CD8 T cell expansion [29]. After interaction with stimulated T cells, monocytes binding anti-CD3 may express additional costimulatory molecules and cytokines as well, generating a more complex costimulatory environment than an inert antibody-coated bead. Whatever the specific signaling events, the adverse impact of beads on activated CD8 cells can not be simply ameliorated by reducing the concentration of anti-CD3 on the bead surface or by the presence of irradiated autologous MNCs at the time of restimulation.

The propensity of anti-CD3/CD28 beads to damage previously activated CD8 T cells has important implications. A second round of stimulation with anti-CD3/CD28 beads is a risky strategy for enhancing the yield of antigen-specific cells. Any increase in absolute cell number must be balanced against damage to surviving cells and unwanted pruning of the repertoire from AICD before expansion. Equally important, if cells activated physiologically behave like in vitro stimulated cells, T cells responding to antigen in vivo (for example tumor-specific CD8 T cells in tumor infiltrating lymphocytes preparations or antiviral CD8 T cells retrieved from the blood of a patient with active viral infection) may be vulnerable to bead-induced AICD or growth retardation during even an initial round of ex vivo stimulation. If so, bead exposure may selectively damage or destroy an important subset of harvested T cells. For this reason, stimulation using soluble anti-CD3 plus MNCs may deserve greater consideration as an alternative approach for expanding of CD8 cells in situations where a desired antigen-specific cell subset may have been "preactivated" in vivo.

We also noted significant phenotypic differences between the CD8 T cells expanded in response to beads and soluble anti-CD3. Consistent with most [22,30,31], but not all [32] prior studies of in vitro stimulated T cells bead and soluble anti-CD3 treated CD4 cells and bead-treated CD8 cells assume predominantly an effector or effector memory phenotype, markedly downregulating CD45RA and CCR7 by day14 post stimulation. By contrast, a subset of anti-CD3 treated CD8 cells retained CD45RA, CD27, and, to lesser extent, CCR7 and CD28 expression at day 14. By studying purified CD8 subset cells, we could establish that these phenotypically distinctive cells were derived from the naïve CD8 T cell subset. The pattern of strong CD45RA expression in cultured CD8 T cells, is often associated with terminally differentiated effectors [22], but based on their derivation from naïve cells, the preservation of CD27, and CCR7 (at week 2 with gradual disappearance by week 3), in the absence of CD57 expression, or rapid intracellular cytokine production in response to restimulation, we suggest these cells evolve towards a phenotype analogous to the CD45RA+, CCR7-, CD27+ CD8 T cells noted previously in human blood [25]. This subset has been shown to have a proliferative history and effector function intermediate between naïve and effector memory cells.

The explanation for these differences in phenotypic evolution of bead and soluble anti-CD3 stimulated cells remains uncertain. Since CD45RA expression can be reversibly downregulated in response to T cell receptor stimulation [33], reduced expression of this antigen could, in part, be explained by the prolonged duration of bead-mediated stimulation noted in these and prior studies [6]. The more prolonged impact of bead stimulation could also contribute to reduced CD27 expression by enhancing and sustaining expression of CD70, a natural ligand, which can downregulate CD27 expression on stimulated T cells [34]. It is unlikely however, that such reversible effects could fully explain CD45RA and CD27 loss. Neither CD45RA nor CD27 were re-expressed on bead-treated cells even after several weeks after bead removal suggesting more durable differences in CD8 T cell expression. Such divergence would not be surprising since differences in the quality, magnitude, and duration of T cells signaling have been well documented to influence T cell differentiation [35].

The functional properties of the CD45RA+ CD8 cells produced during anti-CD3 mediated expansion merit further study. Derived from naïve cells, these relatively "young" cells retain expression of both CCR7, a crucial receptor in lymphoid trafficking to central lymphoid tissues for several weeks, and CD27, an antigen positively associated with T cell proliferation in vitro and engraftment after adoptive transfer in vivo [34,36] for even longer despite substantial expansion. Cells with these properties could have an advantage in trafficking into and proliferating within central lymphoid tissues in vivo compared to bead treated cells expressing an effector phenotype. On the other hand, although these cells do express more CD27 and CCR7, they also often express less CD28 than comparable bead-treated cells, and CD28 expression in some settings has been a surrogate marker for proliferative potential and telomerase expression [37,38]. Clearly, additional in vitro and ultimately in vivo function studies addressing the homing and proliferative capacity of this subset would be needed to accurately assess its properties.

Anti-CD3/CD28 coated beads and soluble anti-CD3 have each been used clinically to expand cells for adoptive transfer with promising results in some settings [8-11,13-15,19]. While beads are extremely effective in stimulating CD4 T cell expansion and in generating bioactive effector cells, the current studies raise the possibility that soluble anti-CD3 may be a safer reagent for expanding CD8 cells which may have been recently antigen-stimulated. The findings also suggest this approach may be more effective in preserving expression of functionally important surface antigens such as CCR7 and CD27 on naive T cells during expansion.

While these studies have focused broadly on the expansion and phenotype of CD4 and CD8 T cell populations, in practical applications it is the ability of any given method to expand functionally active, antigen-specific T cells directed against a defined target pathogen or tumor that is most critical. Evaluating efficacy in achieving this goal is more complicated particularly given the potential phenotypic heterogeneity of antigen-specific populations. Whereas naturally occurring melan A-responsive CD8 T cells are predominantly naïve in phenotype [39], EBV-specific CD8 cell populations contain a high proportion of effector memory cells, and CMV-specfic responses are often dominated by effector CD8 T cells [40,41]. These differences in phenotype and biology may substantially shape how each antigen-specific population respond to any given expansion protocol. Future studies systematically comparing the efficacy of anti-CD3/CD28 beads, soluble anti-CD3, and potentially other expansion protocols in expanding defined populations of antigen-specific naïve, memory, and effector T cells could be helpful in beginning to develop more nuanced guidelines for selecting the best cell expansion method for any particular clinical settings.

Looking to the future, with growing interest in using adoptive transfer to treat human disease, many promising new approaches are under active study for improving T cell expansion. These include the use of other cytokines as well as or instead of IL2 as growth factors, tumor-derived cell lines engineered to express bioactive costimulatory molecules and cytokines as accessory cells [42], and retroviral or lentiviral vectors coding chimeric antigen receptors to confer defined antigenic specificity on T cells [42-44]. In judging the impact of these newer approaches and cell products, the current studies defining the in vitro performance characteristics of "standard" expansion protocols hopefully may serve as a useful "measuring stick" with which to judge the value of new approaches for stimulating and restimulating human T cells.

## Conclusions

Anti-CD3 in the presence of Fc receptor positive accessory cells, is an effective method for expanding CD4 and CD8 T cells which could have potential advantages over anti-CD3/CD28 coated beads in expanding CD8 T cells which may have been recently activated or antigen-exposed in vitro or in vivo without provoking antigen induced cell death. This method also may be advantageous in maintaining CCR7 expression on expanding CD8 cells in settings where central lymphoid trafficking of adoptively transferred CD8 cells may be particularly desireable.

## List of Abbreviations

AICD: activation-induced cell death; Anti-CD3/CD28 beads-beads coated with anti-CD3 and anti-CD28; CFSE: Carboxyfluorescein succinimidyl ester; MNCs: mixed mononuclear cells; REP: Rapid Expansion Protocol.

## Competing interests

The authors declare that they have no competing interests.

## Authors' contributions

YL performed studies, participated in their design, and reviewed the manuscript. RK participated in the design of the studies, performed the statistical analysis and wrote the manuscript.

Both authors read and approved the final manuscript.
